# Analysis of Epilepsy Treatment Strategies Based on an Astrocyte–Neuron-Coupled Network Model

**DOI:** 10.3390/brainsci15050465

**Published:** 2025-04-27

**Authors:** Jianing Lan, Rong Wang

**Affiliations:** School of Science, Xi’an University of Science and Technology, Xi’an 710600, China; 22201106044@stu.xust.edu.cn

**Keywords:** neurodynamics, epilepsy, astrocytes, epilepsy treatment, stimulation regulation, phase-locking value, surgical resection

## Abstract

**Background/Objectives**: Epilepsy is a common neurological disorder that not only severely impacts patients’ health but also imposes a significant burden on families and society. However, its pathogenesis remains unclear. Astrocytes play a crucial role in epileptic seizures and may serve as potential therapeutic targets. Establishing a network model of epileptic seizures based on the astrocyte–neuron cell coupling and the clinical electroencephalographic (EEG) characteristics of epilepsy can facilitate further research on refractory epilepsy and the development of treatment strategies. **Methods:** This study constructs a neuronal network dynamic model of epileptic seizures based on the Watts–Strogatz small-world network, with a particular emphasis on the biological mechanisms of astrocyte–neuron coupling. The phase-locking value (PLV) is used to quantify the degree of network synchronization and to identify the key nodes or connections influencing synchronous seizures, such that two epilepsy treatment strategies are proposed: seizure suppression through stimulation and surgical resection simulation therapy. The therapeutic effects are evaluated based on the PLV-quantified network synchronization. **Results:** The results indicate that the desynchronization effect of random noise and sinusoidal wave stimulation is limited, while square wave stimulation is the most effective. Among the four surgical resection strategies, the effectiveness is the highest when resecting nodes exhibiting epileptic discharges. These findings contribute to the development of rational seizure suppression strategies and provide insights into precise epileptic focus localization and personalized treatment approaches.

## 1. Introduction

Epilepsy is a recurrent, transient brain dysfunction syndrome characterized by sudden episodes of abnormal electrical activity in the brain, leading to generalized convulsion [1,2]. Globally, epilepsy affects approximately 50 million individuals, making it the second most prevalent neurological disorder after stroke [3,4]. The recurrent seizures and the necessity for long-term medication place a substantial psychological and physiological burden on patients [4,5], often leading to severe social stigma, an increased risk of psychiatric disorders such as autism, and even mortality [6,7]. Traditionally, astrocytes were believed to serve only as structural support for neuronal networks and as an energy supply for neurons [8]. However, astrocytes constitute approximately 50% of brain volume and experimental findings have demonstrated that astrocytes can respond to neurotransmitters and provide feedback signals to neurons, thereby regulating synaptic transmission and neuronal excitability [9,10,11]. Current evidence suggests that astrocytes participate in neuronal discharges through the tripartite synapse, either directly or indirectly modulating chemical synaptic processes, and its dynamics are primarily governed by intracellular Ca^2+^ oscillations [12]. When Ca^2+^ concentrations exceed a critical threshold [13], astrocytes release glutamate via exocytosis, thereby modulating neuronal firing activity [14]. Hypothermic treatment has been demonstrated to effectively suppress epileptic seizures [15]. It reduces presynaptic neurotransmitter release and induces the loss of postsynaptic dendritic spines [16]. Additionally, hypothermia inactivates voltage-gated ion channels and slows down metabolic processes, thereby contributing to seizure suppression [17]. In 2016, Soriano et al., based on the neural mass model (NMM), revealed that hypothermia suppresses epilepsy by reducing both the mean and variance of the neuronal population firing threshold distribution [16]. This discovery has fundamentally altered the understanding of brain function, highlighting astrocytes’ role not only in information processing but also in the pathophysiology of neurological disorders such as epilepsy [18].

Traditional antiepileptic drugs (AEDs), such as γ-aminobutyric acid (GABA) modulators, function by enhancing GABAergic activity at chemical synapses to suppress neuronal firing [19]. Huneau et al. discovered that a gradual reduction in GABA inhibition leads to neuronal hyperexcitability, thereby triggering epileptic activity [20]. Similarly, Chen et al. demonstrated that heterogeneity in GABA_A- and GABA_B-mediated modulation within the thalamic reticular nucleus and relay nucleus circuits significantly influences epileptic discharges [21]. However, approximately one-third of epilepsy cases remain pharmacoresistant [22,23]. Neuromodulation is a neurosurgical approach that regulates neural signal transmission within the nervous system through implantable or non-implantable techniques using physical (e.g., electrical, magnetic, optical) or chemical interventions while maintaining the reversibility of brain tissue functions [24]. Electrical pulse stimulation can facilitate the transition from epileptic states to normal conditions [25]. In fact, due to the high target specificity, reversibility, and long-term therapeutic effects of deep brain stimulation (DBS), neuromodulation based on electrical stimulation has become a key focus in research on drug-resistant epilepsy [26]. A substantial body of electrophysiological and clinical evidence indicates that specific electrical stimulation protocols can successfully control or terminate epileptic seizures. In recent years, electrical stimulation for the treatment of drug-resistant epilepsy has garnered significant attention [19]. Berenyi et al. demonstrated that closed-loop transcranial electrical stimulation reduces spike–wave discharges in rodent epilepsy models [27]. Durand et al. [28] found that applying sinusoidal electrical stimulation at 50 Hz to the hippocampal CA1 region significantly suppressed seizure activity [29]. However, astrocytes and neurons form intricate network structures, and neuromodulation primarily targets neurons but is inevitably modulated by astrocytic activity. Studies have shown that high-frequency electrical stimulation not only directly induces Ca^2+^ fluctuations in astrocytes but also enhances intercellular Ca^2+^ signal propagation among astrocytes [30]. Therefore, research on the effects of electrical stimulation on astrocyte–neuron-coupled circuits remains limited. Investigating the impact of electrical stimulation on astrocyte–neuron-coupled dynamics is essential for elucidating the underlying mechanisms of epilepsy treatment and serves as a crucial factor in advancing neuromodulation technologies.

At present, surgical treatment has gradually become an important option for patients with drug-resistant epilepsy. Surgical procedures such as temporal lobe resection, vagus nerve stimulation (VNS), and corpus callosotomy have been widely performed in major medical centers across the country [31]. The indications and techniques for epilepsy surgery continue to expand and innovate, including advancements such as laser ablation and DBS [32]. Recent studies have also explored laser interstitial thermal therapy (LiTT) and stereotactic radiosurgery (SRS) [33], whose applications have significantly enhanced the safety, precision, and efficacy of surgical interventions. However, a substantial proportion of patients remain refractory to current therapeutic approaches, with some exhibiting complete drug resistance. As evidenced by the current state of research, astrocytes play a crucial role in drug-resistant epileptic seizures, while the primary clinical treatment strategies for drug-resistant epilepsy include electrical stimulation and surgical intervention.

Here, based on the fact that Ca^2+^ oscillations within astrocytes can induce the exocytotic release of glutamate, thereby triggering epileptic discharges in neurons, we establish an astrocyte–neuron network coupling model driven by Ca^2+^ oscillations. This model is further extended to construct an epilepsy network model involving astrocytes. We investigate the network dynamics of epileptic seizures to gain a deeper understanding of the dynamical mechanisms underlying epileptic discharges. Additionally, we explore stimulation-based regulation and surgical interventions for epilepsy, providing theoretical guidance for the suppression of refractory and drug-resistant epilepsy.

## 2. Materials and Methods

### 2.1. Epileptic Seizure Network Model

**Figure 1:**
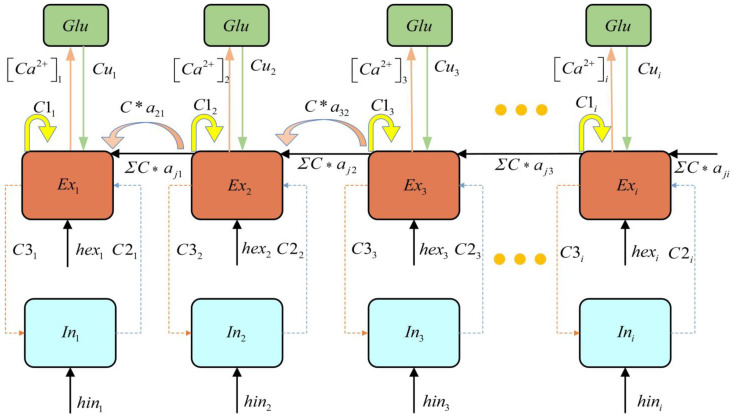
Schematic diagram of epileptogenic network connectivity.

Roberto et al. [34] proposed a tripartite synapse dynamic model based on the coupling between presynaptic and postsynaptic neurons and astrocytes. By employing mean-field approximation theory, they demonstrated that astrocyte populations can regulate the response of neuronal clusters to external stimuli [34]. Therefore, establishing a coupled dynamic model of the astrocyte–neuron network is instrumental in integrating clinical EEG data to investigate the mechanisms by which astrocytes modulate epileptic seizures. Based on the linear relationship between Ca^2+^ concentration and the firing rate of neuronal clusters [35], the glutamate oscillation model of astrocyte populations [36], and the epileptic discharge model coupling excitatory and inhibitory neuronal clusters [37], we establish an epilepsy network model, as expressed in Equation (1). The schematic representation of the epileptogenic network connectivity is shown in Figure 1.

(1) $$\begin{array}{l} \left\{\begin{array}{l} \frac{dEx_{i}}{dt}=\tau_{ex_{i}}\left(h_{ex_{i}}-Ex_{i}+C1_{i}f[Ex_{i}]-C2_{i}f[In_{i}]+\sum_{j=1,i=1,j\neq i}^{N}Ca_{ij}f[Ex_{j}]+Cu_{i}f[I_{i}^{\mathrm{astro}}]\right)\\ I_{i}^{\mathrm{astro}}=10\lambda_{i}[Glu{]}_{i}\\ \frac{dIn_{i}}{dt}=\tau_{in_{i}}\left(h_{in_{i}}-In_{i}+C3_{i}Ex_{i}\right) \end{array}\right.\\ \left\{\begin{array}{l} {\left[{\mathrm{Ca}}^{2+}\right]}_{i}=k\cdot f_{i}\\ \mu \frac{d{\left[Glu\right]}_{i}}{dt}=\left\{\begin{array}{ll} -{\left[Glu\right]}_{i}+Z_{i}-\kappa h_{i}, & {\left[{\mathrm{Ca}}^{2+}\right]}_{i}>{\left[{\mathrm{Ca}}^{2+}\right]}_{th}\\ -{\left[Glu\right]}_{i}-\kappa h_{i}, & {\left[{\mathrm{Ca}}^{2+}\right]}_{i}\leq {\left[{\mathrm{Ca}}^{2+}\right]}_{th} \end{array}\right.\\ \eta \frac{dh_{i}}{dt}=-h_{i}+[Glu{]}_{i}\\ Z_{i}=\left\{\begin{array}{c} k\cdot f_{i}-{\left[{\mathrm{Ca}}^{2+}\right]}_{th}\\ 0 \end{array}\right.\begin{array}{c} ，\\ ， \end{array}\begin{array}{c} k\cdot f_{i}-{\left[{\mathrm{Ca}}^{2+}\right]}_{th}>0\\ k\cdot f_{i}-{\left[{\mathrm{Ca}}^{2+}\right]}_{th}\leq 0 \end{array} \end{array}\right. \end{array}$$

Here, $Ex_{i}$ represents the excitatory neurons, $In_{i}$ represents the firing activity of inhibitory neurons, $I_{i}^{\mathrm{astro}}$ represents astrocyte current, $h_{i}$ is the recovery variable, [Glu] represents the glutamate concentration in astrocytes, [Ca^2+^]*_th_* denotes the threshold calcium concentration. κ describes the coupling coefficient between glutamate concentration and the recovery variable. μ and η are time-dependent constants, Z represents the difference between the Ca^2+^ concentration and the threshold [Ca^2+^]*_th_*. $h_{ex_{i}}$ and $h_{in_{i}}$ are input parameters, $\tau_{ex_{i}}$ and $\tau_{in_{i}}$ are time scale parameters. $C1_{i}$, $C2_{i}$, $C3_{i}$, and $Cu_{i}$ are connectivity parameters. $\lambda_{i}$ denotes the feedback strength of astrocytes to the neuronal population. The sigmoid activation function is defined as: $f\left[x\right]=1/\left(1+\varepsilon^{-x}\right)$, where, x = $Ex_{i}$, $Ex_{j}$, $In_{i}$, $I_{i}^{astro}$, ε = 1000. The simulation step size is set to 0.05 ms, with a total duration of 100 ms. The epilepsy network parameters are set as: $C1_{i}=1$, $C2_{i}=15$, $C3_{i}=6.1$, $Cu_{i}=6$, $\tau_{ex_{i}}=1.97$, $\tau_{in_{i}}=3.0$. Input parameters: $h_{ex_{i}}=-0.5$, $h_{in_{i}}=-1.4$. The model employed in this study is developed based on the Refs. [38,39], in which the dimensionless formulations were adopted, and thus our model is dimensionless Additionally, extensive simulations were conducted to ensure the robustness of the research findings beyond a single example. The parameter range was determined based on the discharge pattern to ensure the emergence of oscillatory dynamics in the system.

**Figure 2:**
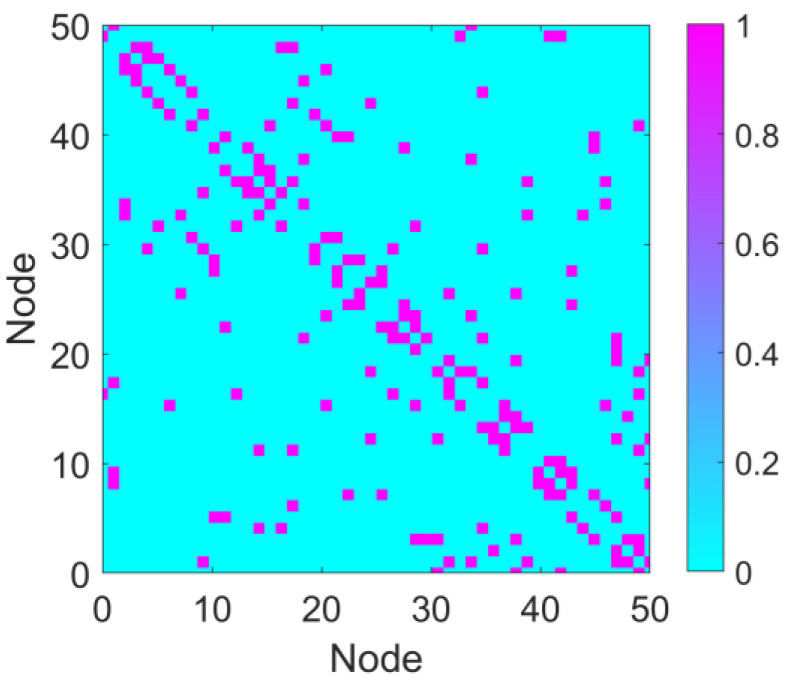
Connectivity matrix. The connectivity matrix of the network with parameters N = 50, N0 = 2, and *p* = 0.5. In the matrix, purple regions represent connections (value = 1), and cyan regions indicate the absence of connections (connection strength = 0).

The astrocyte–neuron-coupled network primarily focuses on the cell–cell coupling mechanisms observed in biology. The network topology does not strictly exhibit scale-free properties but is instead constructed based on specific biological assumptions or experimental data (e.g., random networks, small-world networks). To simulate the topological characteristics of the neuronal network, the connections between neurons were constructed based on the Watts–Strogatz small-world network model. Due to its high clustering coefficient and short path length, the small-world network model effectively captures the structural properties of brain neural networks. The number of nodes in the network, N, the initial number of neighbors N0 (i.e., the maximum number of connections for each node), and the rewiring probability p are defined. We set N = 50, N0 = 2, and *p* = 0.5. The resulting small-world network is represented by the connection matrix ***a***, where *a_ij_* = 1 indicates a connection between node *i* and node *j*, and *a_ij_* = 0 denotes no connection between the two nodes. The network construction is implemented using MATLAB (Matlab r2022a), utilizing the Watts–Strogatz function to generate the network, and the resulting matrix is converted into a symmetric connection matrix to ensure undirected connections. The generated connection matrix ***a*** is an N × N symmetric matrix. The visualization of the connectivity matrix is shown in Figure 2.

### 2.2. Phase Locking Value and Coefficient of Variation

This study employs the Phase Locking Value (PLV) quantification model derived from the Hilbert transform to measure the phase synchronization of the dominant frequency in the model output [40]. PLV is an effective metric for phase synchronization, capable of capturing the consistency of phase relationships between different neurons and reflecting the degree of network synchronization. PLV is defined by Equation (2):

(2) $${\mathrm{PLV}}_{ij}=\left|\frac{1}{T}\sum_{t=1}^{T}e^{j\Delta \phi_{ij}(t)}\right|$$

Here, $\Delta \phi_{ij}(t)$ represents the instantaneous phase difference between any two nodes, *i* and *j*, in the network.

The Hilbert transform function provided by MATLAB is used to compute the complex analytic signal for each node and extract the instantaneous phase. An instantaneous phase difference matrix is constructed for all pairs of nodes. The instantaneous phase difference for each pair of nodes is exponentiated and time-averaged to obtain the PLV matrix of the network. Finally, by analyzing the PLV matrix, the phase synchronization characteristics between neurons in the network are revealed. Regions with high PLV values indicate sub-networks with stronger synchronization properties, thus providing a quantitative basis for understanding network synchronization phenomena associated with epileptic seizures and laying the foundation for developing synchronization-based treatment strategies for epilepsy.

The coefficient of variation (CV) is a statistical metric used to quantify the degree of dispersion within a dataset. It is defined as the ratio of the standard deviation to the mean and is typically expressed as:

(3) $$CV=\frac{\sigma }{\mu }$$

Here, σ represents the standard deviation, reflecting the extent of variability within the data, and μ denotes the mean, indicating the central tendency of the dataset.

CV is particularly useful for comparing the relative dispersion of different datasets, especially when their means differ but an assessment of variability is required [41]. A higher CV value indicates greater fluctuations in network synchrony, suggesting that significant instability persists after surgical treatment, leading to a less favorable outcome. Conversely, a lower CV value signifies a more stable network synchrony post-surgery, corresponding to a better therapeutic effect. By comparing the CV values of different surgical strategies, the effectiveness of each approach in reducing epilepsy network synchrony can be quantitatively assessed, providing a basis for optimizing surgical treatment plans.

### 2.3. Stimulation-Controlled Network Model

A stimulation control strategy is proposed to address the synchronization and dynamic behavior of complex dynamic networks. The fundamental principle of this strategy is to apply directional stimuli to a few key nodes within the small-world network in order to effectively control the network’s synchronization and dynamic behavior. Previous research has identified certain nodes that play crucial roles as hubs within the network. Therefore, the selection of key nodes is based on their high centrality or high synchronization indices (e.g., PLV). The stimulation signal regulates the dynamics of these nodes, influencing their interactions with neighboring nodes and thereby achieving state regulation of the entire network.

A stimulation strategy based on the PLV index was then designed. This strategy involves selecting a small number of nodes with high PLV values in the network for stimulation and regulating their dynamic behavior to effectively control focal epileptic seizures. The study focuses on the impact of different stimulation signals and the proportion of stimulated nodes on the treatment of intermittent seizure states, with the objective of bringing the epileptic network into a healthy state. To achieve this goal, the method proposed in reference [42] is adopted, wherein the stimulation signal is applied to excitatory group nodes. The dynamics of the stimulated nodes under the influence of the stimulation can be represented by the following differential equation:

(4) $$\frac{dEx_{i}}{dt}=\tau_{ex_{i}}\left(\begin{array}{l} h_{ex_{i}}-Ex_{i}+C1_{i}f[Ex_{i}]-C2_{i}f[In_{i}]\\ +\sum_{j=1,i=1,j\neq i}^{N}Ca_{ij}f[Ex_{j}]+Cu_{i}f[I_{i}^{\mathrm{astro}}] \end{array}\right)+S{\left(t\right)}_{i}$$

Here, *S*(*t*) represents an external stimulus which is typically delivered via electrical stimulation devices, such as deep brain stimulation (DBS) by applying voltage. The stimulated nodes correspond to the locations where electrical stimulation is applied. All parameters are the same as those in the node combination network of Section 2.1. The discharge patterns of the nodes in the network are defined as follows: the network consists of 50 nodes, which are ranked in descending order based on their degree. The top 19 nodes, with the highest degrees, are assigned discharge mode 2. The next 11 nodes, ranked 20th to 30th in terms of degree, are assigned discharge mode 3. The remaining 20 nodes, with the lowest degrees, are assigned discharge mode 1.

### 2.4. Surgical Resection Local Node Network Model and Treatment Strategy

Abnormal discharges in epilepsy typically originate from key nodes in specific brain regions, and the discharge patterns of these nodes significantly influence brain network dynamics. Surgical resection is one of the major methods for treating refractory epilepsy. In this study, a brain network model is constructed based on the small-world network. The discharge patterns of the nodes are categorized by calculating the degree distribution of PLV, and the key nodes or connections that most significantly affect synchronized seizures are identified. The adjacency matrix is updated according to different resection strategies. Through neuronal time series simulations, the dynamic changes in node discharge behaviors and glutamate concentrations are computed to evaluate the impact of different surgical resection strategies on brain network dynamics. In Pattern 1, neuron populations are in a resting state and are not considered in the resection.

The node discharge patterns in the network are set as follows: the network consists of 50 nodes, and after sorting the nodes by degree in descending order, the top 20 nodes (with the highest degree) are set to discharge mode 2. The next 8 nodes (ranked 21st to 28th by degree) are set to discharge mode 3. The 29th and 30th nodes are set to discharge mode 4, while the remaining 20 nodes are set to discharge mode 1.

Based on the physiological mechanisms of surgical treatment, different local node resection network models are established, and a discharge pattern-based node grouping resection comparison plan is designed as follows:

Plan 1: Resection of nodes with discharge pattern 2: Nodes with discharge pattern 2 typically exhibit high-frequency, large-amplitude, and strongly synchronized discharge behaviors. In this plan, priority is given to the resection of nodes exhibiting this discharge pattern within the network to investigate their regulatory effects on the activity of remaining network nodes and glutamate concentration fluctuations.

Plan 2: Resection of nodes with discharge pattern 3: Nodes with discharge pattern 3 display intermittent epilepsy discharges characterized by moderate frequency and smaller amplitude.

Plan 3: Resection of nodes with discharge pattern 4: Nodes with discharge pattern 4 exhibit high-amplitude, high-frequency, and sustained saturation of epileptic discharge.

Plan 4: Resection of all epilepsy-related discharge nodes (patterns 2, 3, and 4): in this plan, all nodes exhibiting epileptic discharge characteristics are resected to simulate the network state after the complete removal of the epileptic focus.

The discharge patterns of the nodes are categorized based on their discharge frequency, amplitude, and synchronization characteristics. The classification of discharge patterns reflects the functional differences in nodes within the network and provides the theoretical basis for the design of resection plans. By resecting nodes with different discharge patterns, the plan gradually evaluates their effects on the network. The final resection plan aims to verify whether the complete removal of abnormal discharge nodes is the optimal strategy for controlling epileptic discharges and restoring dynamic balance. This design serves as a reference for clinical surgical treatment.

**Figure 3:**
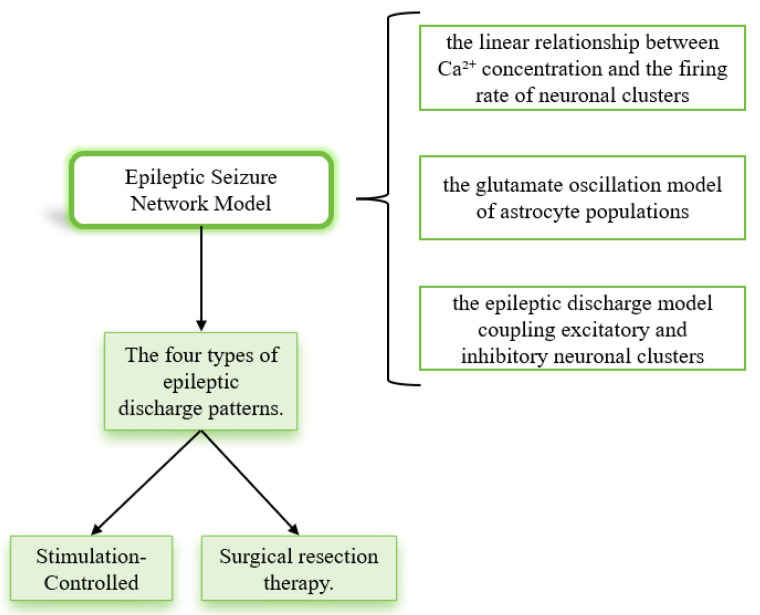
Research flowchart.

The flowchart of the present study is as follows (Figure 3):

## 3. Results

### 3.1. Four Types of Epileptic Discharge Patterns

**Figure 4:**
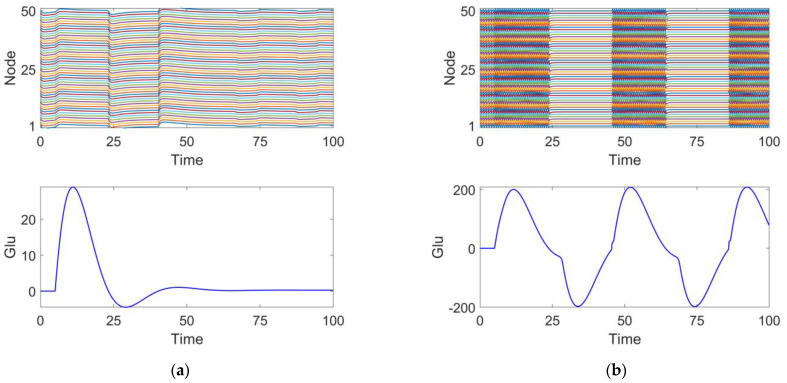

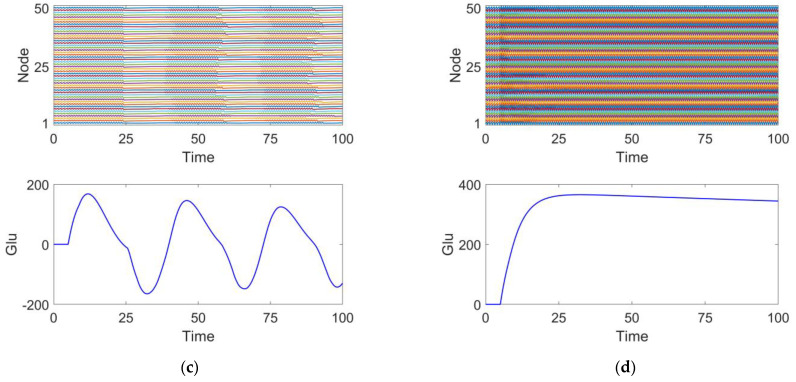
Four discharge modes. Mode 1 (**a**): the neuronal population remains in a relatively inactive state. Mode 2 (**b**): large amplitude and high-frequency oscillations typical of epileptic seizures. Mode 3 (**c**): small amplitude and low-frequency oscillations, representing an intermittent discharge pattern. Mode 4 (**d**): neuronal clusters exhibit saturated discharges.

We investigated the effects of different parameter variations on coupled oscillators with the same timescale in an epileptic seizure neural brain network model. Figure 4 illustrates four types of neurodynamic responses, including the time series of oscillations from all oscillators and changes in glutamate concentration.

At parameter $\tau_{in_{i}}=0.001$, the discharge activity exhibits a relatively uniform distribution, with glutamate release remaining steady around 0, showing no significant periodic changes. This suggests that under this condition, the neuronal population is in a relatively inactive state, which we define as discharge pattern 1, as shown in Figure 4a.

At parameter $h_{ex_{i}}=-0.6$, the discharge activity of the neuronal cluster exhibits clear periodic oscillations. From the excitatory neuron responses, it is evident that the neurons are in a synchronized discharge state, which we define as discharge pattern 2, as shown in Figure 4b.

At parameter $\tau_{ex_{i}}=0.6$, the discharge activity shows intermittent oscillations, indicating that under this condition, neuronal discharge presents complex spatiotemporal dynamics. We define this as discharge pattern 3, as shown in Figure 4c.

At parameter κ=1, the neuronal cluster undergoes saturated discharge, indicating that the neuronal population is in a highly synchronized state. The glutamate concentration initially increases, stabilizing after reaching a peak. We define this as discharge pattern 4, as shown in Figure 4d.

Discharge patterns 2 and 3 both represent spontaneous recurrent epileptic discharges and can be considered intermittent seizure states [43]. Discharge pattern 2 features a large seizure amplitude and high oscillation frequency, while discharge pattern 3 displays a smaller seizure amplitude and lower oscillation frequency. For both typical burst cycles, as shown in Figure 4, when the glutamate concentration exceeds a certain threshold, the brain network model enters an oscillatory phase. Conversely, when the glutamate concentration falls below a threshold, the system’s oscillation cycle ends. By comparing discharge pattern 2 with pattern 3, it is evident that the glutamate concentration fluctuations in discharge pattern 3 are smaller than those in discharge pattern 2. This suggests that the magnitude of glutamate concentration influences the oscillation amplitude and frequency during epileptic seizures.

### 3.2. Analysis of Stimulus-Induced Suppression of Epilepsy

**Figure 5:**
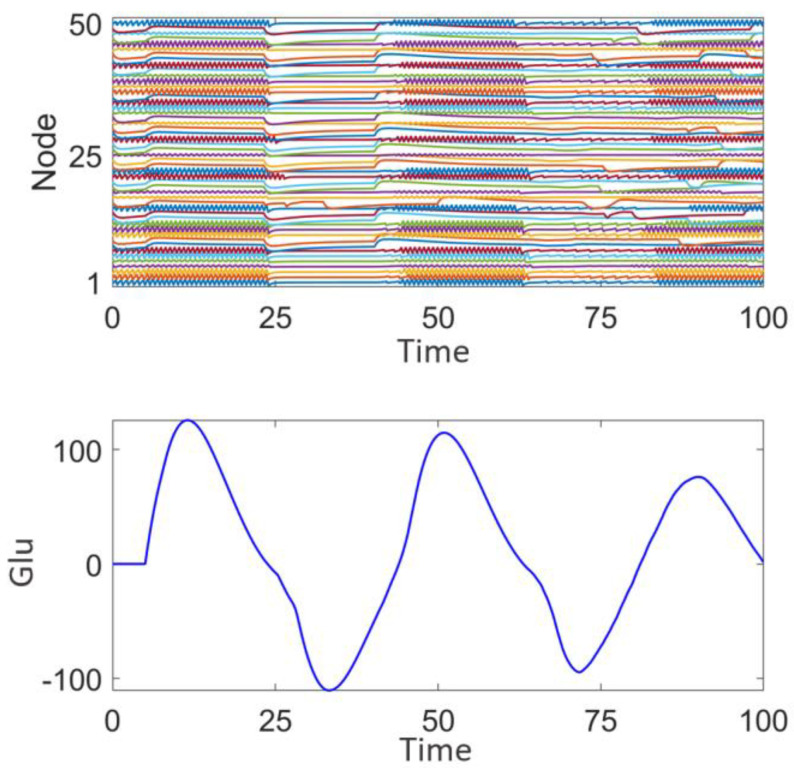
Network response of the epileptic seizure prior to stimulation treatment.

Figure 5 illustrates the network response without the application of stimulation signals. The upper panel displays the discharge patterns of the nodes, while the lower panel shows the fluctuations in glutamate (Glu) concentration. It can be observed that the node discharges exhibit strong synchrony, and the glutamate concentration fluctuates within a certain range. This synchronized discharge is consistent with the characteristics of epileptic seizures [44] and may lead to sustained excitatory transmission, making it difficult to interrupt intermittent epileptic episodes.

**Figure 6:**
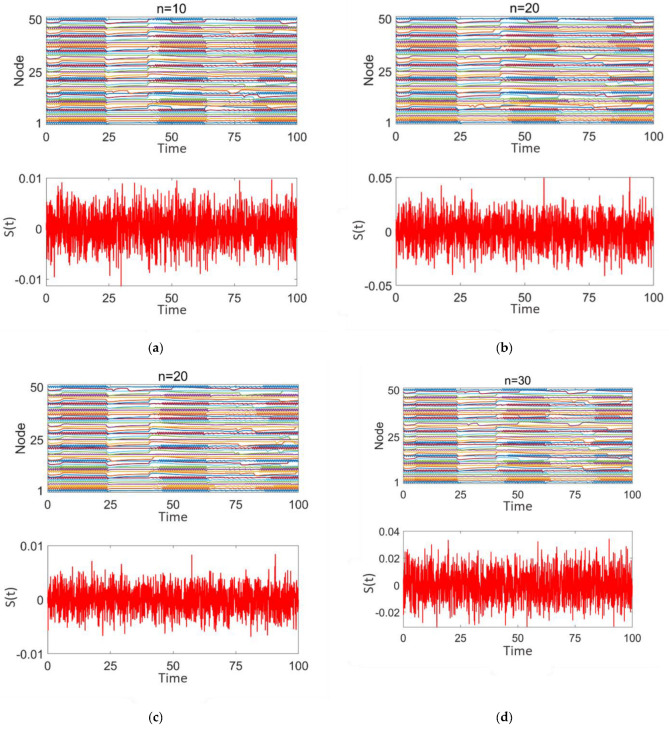
Network response of epileptic seizures after random noise stimulation treatment: (**a**–**d**) correspond to *n* = 10, 20, and 30, respectively. The stimulation intensity is shown in subplot 2 of (**a**–**d**).

Figure 6 demonstrates the impact of random stimulation with varying intensities (z = 0.01, 0.02, 0.05) applied to different numbers of high-degree nodes (*n* = 10, 20, 30) on discharge patterns. Here, z represents the stimulation intensity, and *n* represents the number of high-degree nodes receiving stimulation. Subplot 2 in each panel shows the type and intensity of the random number stimulation applied to these nodes. The analysis is conducted from two perspectives: stimulation intensity and the number of nodes, *n*.

As shown in Figure 6a–d, although there are differences in stimulation intensity across the subplots, the variation in stimulation intensity has minimal impact on the network’s discharge pattern. Strong synchrony persists throughout. When the stimulation intensity remains constant and the number of stimulated nodes increases (as shown in Figure 6a,c) or the number of stimulated nodes remains constant while the stimulation intensity increases (as shown in Figure 6b,c), in both cases, the degree of desynchronization in the network does not improve. The discharge patterns of the nodes continue to exhibit highly synchronized intermittent epileptic discharges, similar to the pre-treatment state. These findings indicate that random stimulation has limited efficacy in promoting network desynchronization.

**Figure 7:**
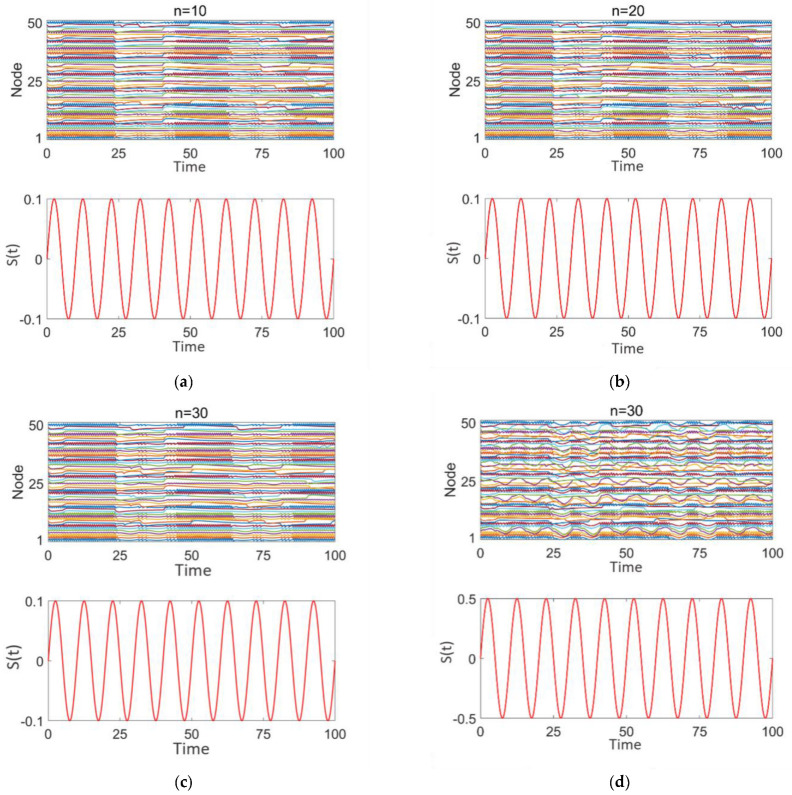
Network response to epilepsy after sinusoidal stimulation treatment: (**a**–**d**) correspond to *n* = 10, 20, 30, 30, respectively. The stimulation intensity is shown in subplot 2 of panels (**a**–**d**).

Figure 7 illustrates the effect of sinusoidal stimulation with varying intensities applied (z = 0.1, 0.5) to different numbers of high-degree nodes (*n* = 10, 20, 30) on discharge patterns. Subplots 1 in panels (a–d) show the discharge patterns of all nodes in the network over time, while subplot 2 presents the time-varying intensity curves of the sinusoidal stimulation signal applied to high-degree nodes.

From Figure 7a–c, it is evident that, when the sinusoidal stimulation intensity is the same (0.1), as the number of stimulated high-degree nodes increases, the discharge behavior of the network nodes continues to exhibit strong periodicity and synchrony, with distinct intermittent epileptic discharge characteristics.

From Figure 7c,d, it is observed that when the number of stimulated high-degree nodes is fixed (*n* = 30), as the stimulation intensity increases (0.1–0.5), the discharge behavior of the network nodes still demonstrates a certain degree of periodicity and synchrony. The intermittent epileptic discharge pattern remains intact and unaffected. However, the discharge intensity of local nodes fluctuates, and the discharge behaviors of some nodes are highly consistent with the sinusoidal stimulation intensity and period. This may be attributed to the nonlinear coupling effects that arise as the stimulation signal propagates through the high-degree nodes in the network.

These findings suggest that the sinusoidal stimulation enters the network through the high-degree nodes and exerts radiative coupling effects on other nodes. However, this stimulation is insufficiently disruptive to the intermittent epileptic discharges, leading the overall network to still exhibit significant periodic synchronization.

**Figure 8:**
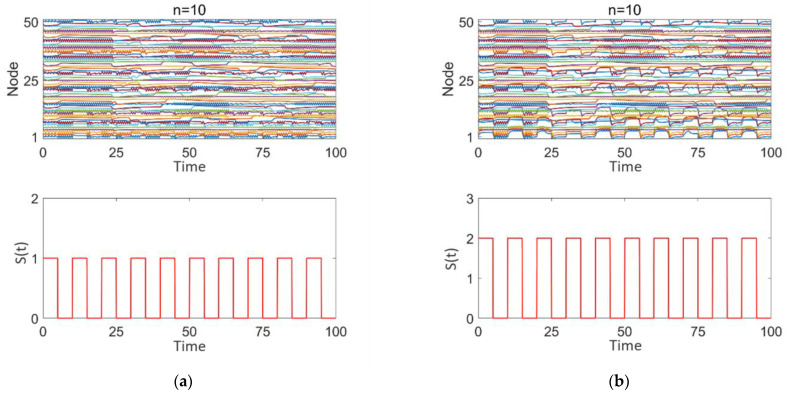

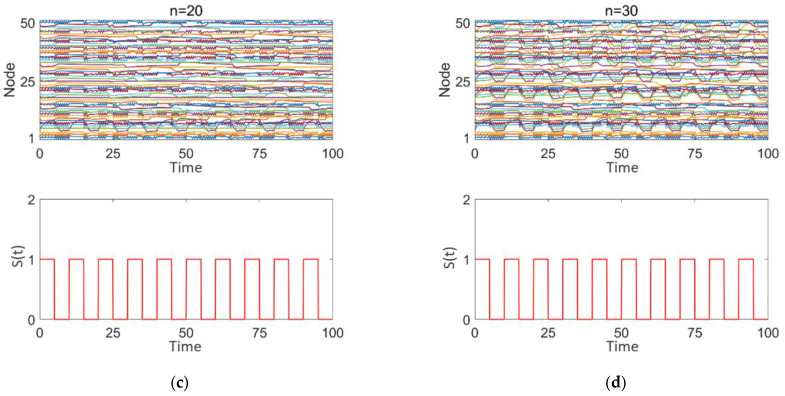
Network response to epileptic seizures after square wave stimulation: (**a**–**d**) correspond to *n* = 10, 10, 20, and 30, respectively. The stimulation intensity is shown in subplot 2 of (**a**–**d**), with the added square wave stimulation intensity being either 0 or positive.

Figure 8a–d illustrates the impact of square wave stimulation with varying intensities (z = 1, 2) applied to different numbers of high-degree nodes (*n* = 10, 20, 30) on the discharge patterns. Subplot 1 shows the dynamic discharge behavior of all nodes in the network over time, and subplot 2 displays the intensity and type (square wave) of the stimulation signal.

As observed in Figure 8a–d, square wave stimulation exhibits a strong disruptive effect on intermittent epileptic discharges. With increasing stimulation intensity (1–2), as seen in Figure 8a,b, intermittent epileptic discharges are significantly suppressed over a large area, and the synchronization between nodes is substantially reduced. However, some regions far from the high-degree nodes may still maintain a certain level of independence. The overall response characteristics of the network undergo significant changes. With an increase in the number of stimulated high-degree nodes (*n* = 10, 20, 30), as shown in Figure 8a,c,d, synchronization within the network significantly decreases, intermittent epileptic discharges are suppressed, and the overall network response shows marked changes. Simultaneously, due to the response of the nodes to the square wave stimulation signal, the discharge patterns between nodes exhibit more complex interactions, with some nodes’ discharge behaviors remaining highly consistent with the square wave stimulation intensity and period.

**Figure 9:**
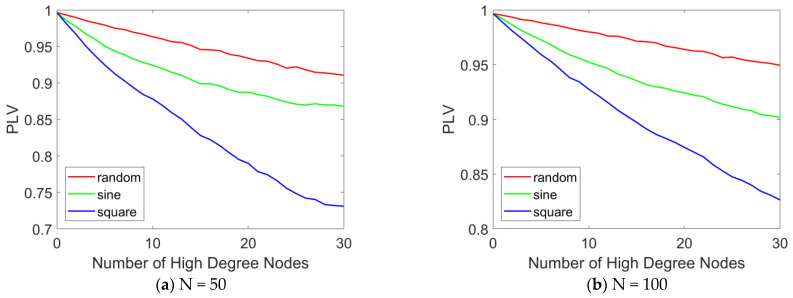
PLV curves with different numbers of stimulated high-degree nodes. The PLV variation curves in the epileptic network model are presented as a function of the number of stimulated high-degree nodes (ranging from 0 to 30) under various stimulation paradigms, including random signals, sinusoidal waves, and square waves. (**a**) N = 50, (**b**) N = 100. When N = 100, the distribution pattern and allocation ratio of nodes in the network remain consistent with those at N = 50. When varying the number of stimulated nodes, the stimulation intensity is set to 0.1.

**Figure 10:**
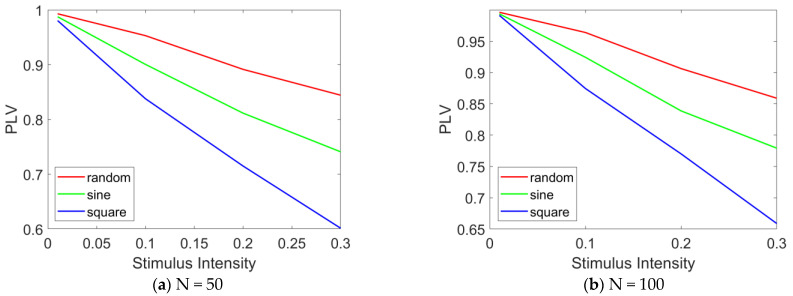
PLV curves under different stimulation intensities. The PLV variation curves in the epileptic network model are presented as a function of the stimulation intensity (ranging from 0 to 0.3) under various stimulation paradigms, including random signals, sinusoidal waves, and square waves. (**a**) N = 50, (**b**) N = 100. When N = 100, and when altering the stimulation intensity, the number of stimulated nodes is fixed at 20.

We employ PLV to quantitatively analyze the degree of network synchronization. Figure 9 and Figure 10 present the variation in PLV in the epileptic network model under different stimulation paradigms (random signals, sinusoidal waves, and square waves) as a function of the stimulated number of high-degree nodes and of the stimulation intensity. The number of high-degree nodes ranges from 0 to 30, while the stimulation intensity varies from 0.01 to 0.3.

Figure 9 illustrates the variation in PLV with the number of stimulated high-degree nodes. The results indicate that as the number of stimulated high-degree nodes increases, the overall PLV of the network changes accordingly. In Figure 9a, when N = 50, under random stimulation, the PLV remains stable at approximately 0.9, suggesting that the network consistently maintains a highly synchronized state. Under sinusoidal stimulation, the PLV slightly decreases but remains around 0.85, indicating that the network still exhibits a high level of synchronization. Under square wave stimulation, the PLV drops below 0.75, but the network still demonstrates relatively high synchronization. A comparative analysis of the PLV across the three stimulation modes reveals that square wave stimulation produces the most effective treatment outcome, while sinusoidal and random stimulations show more limited effects. When N = 100, as shown in Figure 9b, the same conclusion can be drawn: as the number of stimulated high-degree nodes increases, network synchrony decreases. Furthermore, under square wave stimulation, the desynchronization effect is most pronounced.

Figure 10 illustrates the variation in PLV with stimulation intensity. The results indicate that as the stimulation intensity increases, the overall PLV of the network decreases significantly. In Figure 10a, under random stimulation, the PLV decreases to approximately 0.85, suggesting a reduction in network synchronization, although a relatively high level of synchronization is still maintained. Under sinusoidal stimulation, the PLV drops to approximately 0.75, reflecting a further decline in synchronization, while still maintaining a moderately high level. Under square wave stimulation, the PLV falls below 0.6, indicating a significant reduction in network synchronization. A comparative analysis of the PLV across the three stimulation modes reveals that square wave stimulation induces the most pronounced desynchronization effect, followed by sinusoidal stimulation, with random stimulation showing the weakest effect. When N = 100, as shown in Figure 10b, the same conclusion can be drawn: as the stimulation intensity increases, network synchrony gradually decreases. Furthermore, under square wave stimulation, the desynchronization effect is most pronounced.

The results of this study suggest that square wave stimulation may be effective in improving certain desynchronization phenomena in epilepsy treatment, such as early intervention in epileptic seizures. Strong stimulation can drive the entire network but may induce nonlinear coupling effects, leading to local desynchronization. Caution is required to avoid excessive desynchronization effects. These findings provide theoretical support for regulating the dynamic behavior of complex networks through the stimulation of highly connected nodes, while also laying the foundation for further research on the astrocyte–neuron network.

### 3.3. Outcome Analysis of Surgical Resection in the Treatment of Epilepsy

**Figure 11:**
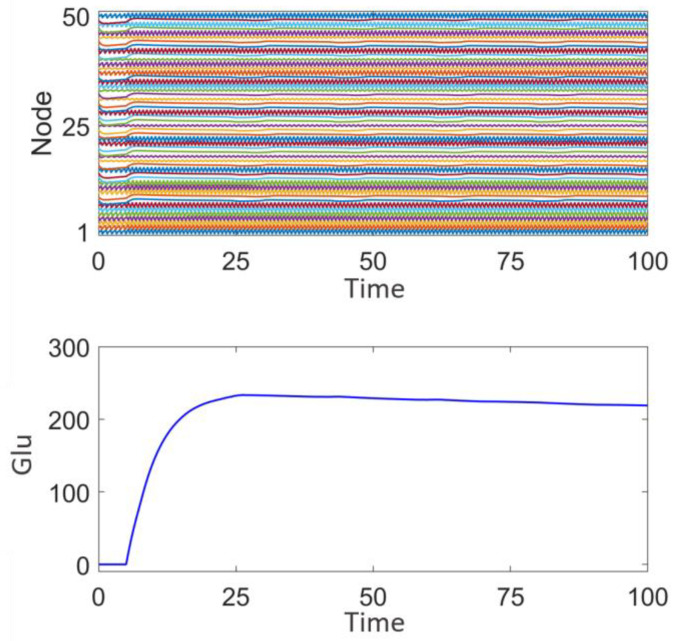
Network response prior to surgical treatment and epileptic focus resection.

Figure 11 shows the discharge activity of a 50-node network prior to surgical treatment. Each node exhibits high-frequency synchronized oscillations, with relatively consistent amplitude. These high-frequency synchronized oscillations are typically associated with epileptic discharges, indicating that multiple nodes in the network are discharging abnormally in a coordinated manner. From the fluctuation of glutamate concentration, it can be observed that the concentration rapidly rises to approximately 300 at the beginning, and then gradually stabilizes. The sustained high level of glutamate concentration suggests the presence of continuous excitation and abnormal discharges within the network, which aligns with the neuronal hyperexcitability observed during epileptic seizures and is consistent with the four distinct discharge mode node combination network model established in this study.

**Figure 12:**
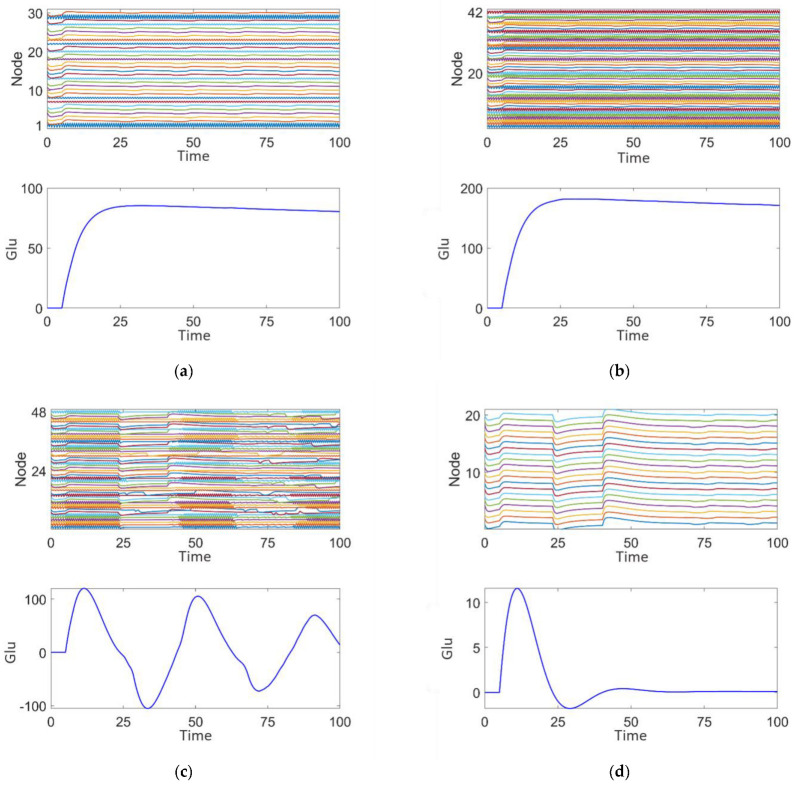
Post-surgical treatment responses of the epileptic network dynamics: (**a**) Plan 1. (**b**) Plan 2. (**c**) Plan 3. (**d**) Plan 4.

Figure 12a illustrates the effect of removing nodes in discharge mode 2. After the removal, the node activity graph shows that the remaining nodes in the network still exhibit a high degree of synchronization, demonstrating saturated high-frequency synchronized oscillations. A slight reduction in the frequency and amplitude of activity is observed in some of the network’s nodes. The glutamate concentration graph indicates that, following the removal of nodes in discharge mode 2, the glutamate concentration is significantly lower than before the surgical treatment, reaching a maximum of 80 and eventually stabilizing. This suggests that nodes with high-frequency, high-amplitude synchronized discharges have a significant impact on the overall network’s glutamate concentration fluctuations. This indicates that the removal of discharge mode 2 nodes affects the overall discharge pattern of the network, but the disturbance to the overall dynamics is limited, and the system still exhibits some degree of synchronization and regularity.

Figure 12b shows the effect of removing nodes in discharge mode 3. After removal, the node activity demonstrates a more regular pattern of saturated high-frequency synchronized oscillations. The glutamate concentration is lower than before surgical treatment, peaking at 180, but still higher than the 80 seen after the removal of discharge mode 2 nodes (Figure 12a). The concentration fluctuations are relatively smaller, indicating that the nodes exhibiting medium-frequency, low-amplitude intermittent epileptiform discharges contribute to maintaining network rhythmicity, but the effect is less significant compared to discharge mode 2.

Figure 12c shows the effect of removing nodes in discharge mode 4. The node activity graph reveals intermittent epileptic oscillations with significantly reduced high-frequency abnormal discharges. The glutamate concentration is significantly lower, peaking at 110, and shows some fluctuation. This indicates that nodes exhibiting persistent saturated epileptic discharges contribute significantly to abnormal fluctuations in both network activity and glutamate concentration. Comparing Figure 12a,c, it can be observed that the glutamate concentration peak in Figure 12a is higher, suggesting that discharge mode 4, characterized by persistent saturated discharges, has the most significant impact on epileptic activity in the network.

Figure 12d illustrates that after the complete removal of nodes associated with discharge modes 2, 3, and 4, the activity of the remaining nodes in the network is significantly reduced, resulting in a highly stable state with almost no epileptiform discharge characteristics. The glutamate concentration graph shows that the fluctuation of glutamate concentration approaches zero, indicating that the complete removal of epileptiform discharge nodes can effectively suppress abnormal glutamate concentration fluctuations and restore network stability.

By comparing the results of the four resection schemes, it is evident that nodes in different discharge modes play distinct roles in network stability and glutamate concentration fluctuations. Removal of nodes in discharge modes 2, 3, and 4 has a significant effect on the regulation of high-frequency synchronized discharges, medium-frequency intermittent discharges, and persistent saturated discharges, respectively. The complete removal of all epileptiform discharge-related nodes (modes 2, 3, and 4) can maximize the restoration of network stability and significantly suppress abnormal fluctuations in glutamate concentration. However, the removal of all epileptic nodes is not realistic in clinical treatment, as it may impact the patient’s basic cognitive functions. These findings provide theoretical support for the precise localization of epileptic foci and surgical treatment strategies.

At the same time, the above analysis indicates that nodes in different discharge modes have significantly different effects on network dynamics. The removal of nodes from specific discharge modes requires a comprehensive consideration of their contribution to overall network stability, avoiding indiscriminate removal that could lead to adverse consequences. Furthermore, the removal of all epileptiform discharge-related nodes may be an effective method to achieve dynamic stability, but in practical application, a balance must be struck between the amount of resection and the protection of the patient’s neural functions.

**Figure 13:**
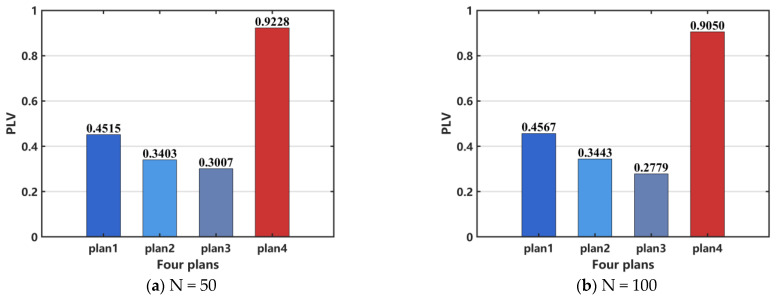
Comparison of network PLV after surgical resection. (**a**) N = 50, (**b**) N = 100. When N = 100, the distribution pattern and allocation ratio of nodes in the network remain consistent with those at N = 50.

We use the PLV to quantitatively analyze the degree of synchronization in the network following surgical resection treatment. Figure 13 illustrates the changes in the PLV of the epilepsy network model under different surgical treatment plans. As shown in Figure 13, the fourth surgical resection plan results in the highest PLV, which may be attributed to the removal of all epileptic discharge nodes, leaving the remaining nodes in a state of synchronized rest, as shown in Figure 12d.

**Figure 14:**
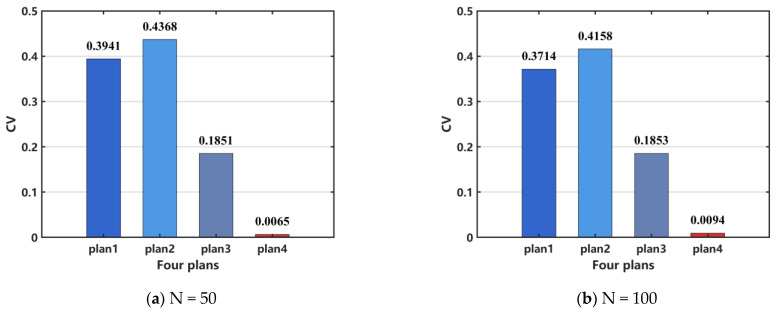
Comparison of network coefficient of variation (CV) after surgical resection. (**a**) N = 50, (**b**) N = 100. When N = 100, the distribution pattern and allocation ratio of nodes in the network remain consistent with those at N = 50.

However, PLV can quantify phase alignment between different signals, but it cannot effectively distinguish between a completely inactive network and a healthy network in a desynchronized state. Additionally, it may not accurately reflect network synchronization in cases of weak signals or high noise levels. Therefore, PLV is unavailable to assess the efficacy of surgical resection. In order to perform a quantitative analysis, we select CV as the indicator to evaluate the effectiveness of the surgical treatment. The results, shown in Figure 14a, indicate that the treatment effectiveness is ranked from the best to the worst as follows: plan 4, plan 3, plan 2, and plan 1. For the case when N = 100, the same conclusion can be drawn, as shown in Figure 14b.

## 4. Discussion

The preferred treatment for drug-resistant epilepsy (DRE) is surgical intervention. However, for patients who are ineligible for surgery or those in whom surgical treatment proves ineffective, DBS serves as a crucial alternative. Effective DBS gradually modulates brain activity, leading to reduced sensitivity and specificity of electrophysiological characteristics and anatomical alterations [45]. However, a single network configuration fails to fully capture the connectivity of individual patients. Therefore, parameter fitting based on each patient’s clinical EEG characteristics or other neuroimaging data, in conjunction with the epilepsy network established in this study, could refine key astrocytic model parameters, enabling personalized treatment [46,47,48,49,50,51,52,53]. Once an accurate network dynamics model is established for an individual patient, their response to stimulation can be predicted, facilitating the development of more effective stimulation strategies. Future research could explore more complex models that incorporate additional factors, such as the heterogeneity of glial cells, their spatial distribution in the brain, and the influence of temperature on glial cell activity. Attention should also be given to the roles of other glial cell types, such as oligodendrocytes and microglia, in epilepsy network dynamics. In this study, we have preliminarily investigated the effects of stimulation treatment in focal epilepsy. Future research could focus on accurately locating the epileptic focus through Granger causality analysis, thereby seeking to maximize therapeutic efficacy [50]. In clinical practice, epilepsy is typically managed through antiepileptic drugs (AEDs), surgical interventions, and neurostimulation therapies. The effectiveness of these treatments often hinges on a precise understanding of the dynamics of seizure activity, including network connectivity and synchrony. Our study explores the impact of different stimulation paradigms on network synchronization, providing a theoretical framework that could inform the development of more targeted therapeutic strategies. For example, square wave stimulation in our model produced the most effective desynchronization, which aligns with the emerging trend in the clinical practice of DBS aimed at reducing excessive synchronization within epileptic networks. A deeper understanding of the specific parameters influencing network synchronization, as highlighted in our study, could contribute to optimizing DBS protocols and improving treatment outcomes for patients.

Although stimulation therapy is less invasive than open brain surgery, DBS still requires the implantation of electrodes into the brain, which introduces risks such as infection, bleeding, and device malfunctions. DBS does not cure epilepsy; rather, it reduces the frequency and severity of seizures in some patients, with its effectiveness varying across individuals. DBS offers a targeted method for modulating brain activity, helping to reduce the synchronization of epileptic networks without affecting the entire brain.

Surgical resection carries inherent risks, including damage to healthy brain tissue, bleeding, infection, and the potential emergence of new neurological deficits, such as memory loss or motor dysfunction. Not all patients are candidates for surgery, especially when the seizure focus cannot be precisely localized or when the epilepsy involves widespread regions of the brain, making surgical resection ineffective. For patients with focal epilepsy where the seizure focus can be accurately identified, surgical resection can be highly effective, significantly reducing seizure frequency and, in some cases, even achieving complete seizure cessation.

## 5. Limitations

There are several limitations to this research. First, we utilized the Watts–Strogatz small-world network model to investigate the impact of coupling strength and network regularity on epileptic network dynamics. Future research could explore more complex network models, incorporating dynamic changes in network structure and considering alterations in network connectivity during epilepsy onset and subsequent recovery phases. Second, this study examined two treatment methods, but future work should focus on developing more targeted and minimally invasive therapeutic strategies, especially for refractory epilepsy. For example, adaptive stimulation strategies based on real-time EEG activity changes could offer more effective epilepsy control while reducing side effects [25]. Furthermore, exploring gene therapies or pharmacological interventions to regulate astrocyte activity [54] could provide new avenues to improve treatment outcomes. Third, this study did not incorporate clinical EEG data to optimize model parameters and remains in the realm of theoretical analysis. Future research could validate and further refine the model by integrating a clinical electroencephalogram (EEG) and other neuroimaging data. Based on clinical EEG data, the model parameters for the ictal and interictal states can be fitted to determine the key parameters of astrocyte–neuron coupling. This laid the foundation for the development of synchronization-based epilepsy treatment strategies and the formulation of personalized epilepsy treatment plans. By combining with patient-specific data, more accurate predictions of epilepsy onset could be made and personalized treatment strategies could be supported. Fourth, while this study preliminarily explored the effects of stimulation treatment in focal epilepsy, future research should focus on more precise optimization of stimulation parameters, such as timing, frequency, and location, to maximize treatment efficacy [55]. Additionally, further investigations into how different types of stimulation (e.g., electrical stimulation, optogenetic stimulation) affect network synchronization and suppression of epileptic seizures [25] could enhance treatment protocols.

## 6. Conclusions

This study constructs the connection relationships between neurons based on the Watts–Strogatz small-world network model and develops a gliocyte–neuron coupling network model (epileptic brain network coupling dynamics model) that emphasizes cell–cell coupling mechanisms in biology. Additionally, two epilepsy treatment approaches and their respective outcomes are presented. First, a stimulation-controlled network model is established, proposing a target-based stimulation regulation strategy. By applying directional stimulation to a few critical nodes (with high PLV values), effective network control is achieved. Second, based on the Watts–Strogatz small-world network model, node discharge patterns are classified, and the network’s degree distribution and synchronization contribution rates are calculated to identify key nodes or connections that affect synchronized seizures. Subsequently, a surgical resection model for localized node networks is constructed, and four different treatment plans are designed. Through neuron temporal simulations, the impact of different surgical resection strategies on brain network dynamics is evaluated. The results indicate that square wave stimulation is more effective in modulating epileptic activity compared to random noise and sinusoidal stimulation. Among the surgical resection strategies, plan 4 demonstrates the highest efficacy, followed by plan 3, plan 1, and plan 2, which exhibits the least effectiveness. However, in practical clinical applications, a balance must be struck between the extent of resection and the preservation of the patient’s neural functions to ensure optimal therapeutic outcomes.

## Data Availability

Data are contained within the article.
